# Prevalence of resistance associated polymorphisms in *Plasmodium falciparum *field isolates from southern Pakistan

**DOI:** 10.1186/1475-2875-10-18

**Published:** 2011-01-28

**Authors:** Najia Karim Ghanchi, Johan Ursing, Mohammad A Beg, Maria I Veiga, Sana Jafri, Andreas Mårtensson

**Affiliations:** 1 Department of Pathology and Microbiology, Aga Khan University, Stadium Road, P.O. Box 3500, Karachi 74800, Pakistan; 2 Malaria Research Lab, Infectious Diseases Unit, Department of Medicine, Karolinska University Hospital/Karolinska Institutet, Retziusväg 10, 171 77 Stockholm, Sweden; 3 Drug Resistance and Pharmacogenetics Group, Institute of Biotechnology and Bioengineering, Centre of Molecular and Structural Biomedicine, University of Algarve, 8500-139 Faro, Portugal; 4 Division of Global Health (IHCAR), Department of Public Health Sciences, Karolinska Institutet, Nobelsväg 9, S-171 77 Stockholm, Sweden

## Abstract

**Background:**

Scarce data are available on *Plasmodium falciparum *anti-malarial drug resistance in Pakistan. The aim of this study was, therefore, to determine the prevalence of *P. falciparum *resistance associated polymorphisms in field isolates from southern Pakistan.

**Methods:**

Blood samples from 244 patients with blood-slide confirmed *P. falciparum *mono-infections were collected between 2005-2007. Single nucleotide polymorphisms in the *P. falciparum *chloroquine resistance transporter (*pfcrt *K76T), multi drug resistance (*pfmdr1 *N86Y), dihydrofolate reductase (*pfdhfr *A16V, N51I, C59R, S108N, I164L) and dihydropteroate synthetase (*pfdhps *A436S, G437A and E540K) genes and *pfmdr1 *gene copy numbers were determined using PCR based methods.

**Results:**

The prevalence of *pfcrt *76T and *pfmdr1 *86Y was 93% and 57%, respectively. The prevalence of *pfdhfr *double mutations 59R + 108N/51R + 108N was 92%. The *pfdhfr *triple mutation (51I, 59R, 108N) occurred in 3% of samples. The *pfdhfr *(51I, 59R, 108N) and *pfdhps *(437G, 540E) quintuple mutation was found in one isolate. *Pfdhps *437G was observed in 51% and 540E in 1% of the isolates. One isolate had two *pfmdr1 *copies and carried the *pfmdr1 *86Y and *pfcrt *76T alleles.

**Conclusions:**

The results indicate high prevalence of *in vivo *resistance to chloroquine, whereas high grade resistance to sulphadoxine-pyrimethamine does not appear to be widespread among *P. falciparum *in southern Pakistan.

## Background

Development and spread of *Plasmodium falciparum *resistance to anti-malarial drugs represents a major threat to global malaria control. In Pakistan, an estimated 500,000 episodes of malaria infection occur annually [1]. The incidence of malaria has markedly increased during the last decade and the relative frequency of *P. falciparum *amongst blood-slide positive malaria infections has increased from 45% in 1995 to 68% in 2006 [2-4], probably due to increasing resistance to commonly used monotherapies.

Chloroquine resistance was first reported from Pakistan in 1984 and was followed by several reports confirming it in Punjab, Afghan refugee camps and areas bordering Afghanistan [5-8]. In agreement with recommendations by the World Health Organization, Pakistan has adopted artemisinin-based combination therapy (ACT) as treatment of choice for uncomplicated *P. falciparum *malaria with artesunate plus sulphadoxine-pyrimethamine as first-line treatment [9]. However, the use of this combination is of some concern as resistance to sulphadoxine-pyrimethamine monotherapy has been reported from western Pakistan [10].

Analyses of molecular markers associated with *P. falciparum *anti-malarial drug resistance can provide important information about levels of sulphadoxine-pyrimethamine resistance. Single nucleotide polymorphisms (SNPs) at codons 51, 59, 108 and 164 in the *P. falciparum *dihydrofolate reductase gene (*pfdhfr*) are well established determinants of pyrimethamine resistance [11]. An initial mutation at codon 108 causes 7-50 fold increase in the *in vitro *inhibitory concentration (IC50). The presence of additional mutations further increase IC50 and the triple mutant (N51I/C59R/S108N) is associated with clinical treatment failure [12,13]. Similarly, mutations at codons 436, 437, 540, 581 and 613 in the *P. falciparum *dihydroptereoate synthase gene (*pfdhps*) are associated with sulphadoxine resistance [14,15].

Other genetic polymorphisms have also been associated with *P. falciparum *drug tolerance/resistance. The K76T amino acid substitution in the chloroquine resistance transporter gene (*pfcrt*) has been shown to be essential for chloroquine resistance, associated with amodiaquine resistance and predictive of treatment failure for both drugs [16,17]. *Pfcrt *76K has been associated with lumefantrine tolerance/resistance and higher IC50 values [18-20]. The *P. falciparum *multidrug resistance gene 1 **(***pfmdr1) *allele 86Y has been associated with chloroquine and amodiaquine resistance and increased chloroquine IC50 values in *P. falciparum *with *pfcrt *76T [21,22]. In a meta analysis *pfmdr1 *86Y was significantly linked to chloroquine and amodiaquine resistance [23]. Conversely, *pfmdr1 *86N has been associated with lumefantrine tolerance/resistance and higher lumefantrine IC50 [20,24,25]. *Pfmdr1 *amplifications are associated with mefloquine resistance in vivo and in vitro, doubled lumefantrine IC50 and reduced sensitivity to artesunate [26].

Scarce data are available on anti-malarial drug resistance among the *P. falciparum *population in Pakistan. The aim of this study was, therefore, to assess the prevalence of *P. falciparum *resistance associated polymorphisms in field isolates from southern Pakistan.

## Methods

### Study setting, participants and ethics

The study was conducted between October 2005 and October 2007 at the Aga Khan University Hospital, a tertiary hospital located in central Karachi, and its established chain of primary health care and diagnostic service centres located in Sindh and Baluchistan provinces, Pakistan. In the study area, malaria transmission peaks during and after the monsoon season that lasts from June to October. Patients with microscopy confirmed *P. falciparum *mono-infection were eligible for enrolment irrespective of age, sex and disease severity.

The study was conducted in accordance with the Declaration of Helsinki and Good Clinical Practice [27]. Informed consent was obtained from all participants or in case of children from their parents/legal guardians. The study was approved by the ethical review committee of Aga Khan University Hospital, Karachi, Pakistan.

### Blood collection and microscopy

Two ml of intravenous blood were collected in an EDTA tube from all patients referred to the laboratory for investigation of malaria infection. For screening purposes a thick blood film was prepared and analysed using Leishman stain according to routine practice. In case of a positive screening result, a thick and thin Giemsa-stained blood film was prepared for confirmation of the presence of malaria parasites and species identification. For all patients with confirmed *P. falciparum *mono-infection the parasite density was assessed by counting asexual parasites against 200 white blood cells (WBC) on the thick film and quantified (parasites/μl) by assuming an average of 8000 WBC per μl blood [28]. All blood slides were examined by experienced microscopists at the clinical laboratory of Aga Khan University Hospital. For quality control, 10% of the blood slides were re-examined by an independent microscopist unaware of the initial result.

From each sample 100 μl of blood was spotted on an FTA^® ^filter paper (Whatman), dried, then put in a separate plastic bag and stored at -80°C. The remaining blood was transferred to cryo-vials and kept frozen at -80ºC until used for DNA extraction. A brief epidemiological and demographic history was also collected from each participant using a structured questionnaire.

### DNA extraction

DNA was extracted using Qiamp DNA mini Kits (Qiagen, USA) from 200 μl of whole blood at Aga Khan University Hospital. The FTA^® ^filter papers were transported to Karolinska Institutet, where DNA was extracted from approximately half of each blood sample (50 μl) using an ABI Prism^® ^6100 Nucleic Acid Prep Station (Applied Biosystems, Fresno, CA). In both cases DNA extraction was according to the manufacturer's instructions. Extracted DNA was stored at -20°C until amplified by PCR.

### Genotyping

*Pfcrt *K76T and *pfmdr1 *N86Y alleles were analysed for all samples at Aga Khan University Hospital. Quality control was done at Karolinska Institutet by genotyping a randomly selected subset of 25% of the samples. The same multiplex PCR-RFLP protocol [29] was used at both laboratories.

SNPs at *pfdhfr *codons 16, 51, 59, 108 and 164, and *pfdhps *codons 436, 437 and 540 were identified by sequencing according to a previously described method [30]. The PCRs were performed at Karolinska Institutet. The products were purified and sequenced commercially (Macrogen Inc. Seoul, Korea). For quality control, the genotype at codon 540 was determined from 25% of the samples using a PCR-RFLP based method [29]. Codon 540 was chosen because of its location towards the end of the amplified segment, where sequencing is less robust. PCR and restriction products were resolved on 2% agarose gels (Amresco, Solon, OH). All gels were stained with ethidium bromide and visualized under UV transillumination (GelDoc^®^, Biorad, Hercules, Ca, USA).

*Pfmdr1 *gene copy number variation polymorphism was determined using real time PCR (ABI Prism^® ^7000 Sequence Detection System) as previously described [26]. All samples were run in triplicate. The clones, 3D7, D10 and K1 were used as single copy calibrators and FCB and Dd2 represented multiple copy controls. *Pfmdr1 *copy numbers were calculated using a comparative threshold method (ΔΔC_t _method) [26]. An amplification of *pfmdr1 *was defined as copy number >1.6. Assays were repeated if the following results were obtained: copy number 1.3-1.6 or Ct value >35 or ΔΔC_t _spread >1.5.

### Power calculation and statistical analyses

This was a descriptive/exploratory study precluding a power calculation of sample size. Data were entered, validated and analysed using SPSS version 16.0. The Sequencher ™ software version 4.6 (Gene Codes Corporation, Ann Arbor, MI) was used to analyse the sequences output using the 3D7 clone sequence obtained from NCBI database (*pfdhfr *Accession # JO4643 and *pfdhps *XM_001349382) as a reference. Allele proportions were calculated as the number carrying a certain allele divided by the number of samples with positive PCR outcome. Mixed infections thus contribute to the prevalence of both alleles.

## Results

### Patients

A total of 244 patients with microscopy confirmed *P. falciparum *mono-infection were enrolled. Baseline demographic data are presented in Table 1.

**Table 1:** Baseline characteristics of enrolled patients.

	All (n = 244)	Karachi (n = 182)	Sindh (n = 52)	Baluchistan (n = 10)
Age *				
≤**5 years**	35 (0.14)	16 (0.09)	17 (0.33)	2 (0.20)
**6-15 years**	39 (0.16)	26 (0.14)	12 (0.23)	1 (0.10)
**>15 years**	170 (0.70)	140 (0.77)	23 (0.44)	7 (0.70)
Sex*				
**Male**	173 (0.71)	137 (0.75)	31 (0.60)	5 (0.50)
**Female**	71 (0.29)	45 (0.25)	21 (0.40)	5 (0.50)
**Parasite density**^**§ **^**(parasites/μl)**	11100 (80-540000)	12720 (80-540000)	7480 (80-126000)	66160 (240-230220)
**Gametocyte carriage**	96 (0.45)	69 (0.43)	23 (0.48)	4 (0.45)

* Number of patients are presented with proportions in brackets

§ Parasite densities were available form 216 patients. Median data are presented with range in brackets.

### *Pfcrt *and *pfmdr1 *SNPs

*Pfcrt *K76T and *pfmdr 1 *N86Y were successfully amplified in 240/244 (98%) samples. Allele proportions are presented in Table 2. *Pfcrt *76T and *pfmdr1 *86Y occurred together in 127/240 (53%) patients. *Pfcrt *76T and *pfmdr1 *86N were observed in 96/240 (40%) of patients. *Pfcrt *76K and *pfmdr1 *86N were found together in 8/240 (3%). PCR amplification failed repeatedly in 4/244 (2%) samples. For that reason these samples were excluded from further PCR analyses (*pfmdr1 *copy number, *pfdhfr *and *pfdhps*)

**Table 2:** Prevalence of *pfcrt *76K, 76T, *pfmdr1 86N and 86Y *alleles in southern Pakistan

	*Pfcrt*				*Pfmdr 1*		
	
	n	76K	76T	76T/K	86N	86Y	86Y/N
**Karachi**	178	16 (0.09)	161 (0.91)	1 (0.006)	73 (0.41)	84 (0.47)	21 (0.12)
**Sindh**	52	0	52 (1)	0	25 (0.48)	20 (0.39)	7 (0.14)
**Baluchistan**	10	1 (0.10)	9 (0.90)	0	6 (0.60)	4 (0.40)	0
**Total**	240	17 (0.07)	222 (0.93)	1 (0.004)	104 (0.43)	108 (0.45)	28 (0.12)

Allele proportions are shown in brackets.

### *Pfmdr1 *copy number

The real-time PCR was successful in 232/240 (97%) samples. A single *pfmdr1 *gene was found in 231 samples. One sample had 2 copies of the *pfmdr1 *gene and carried the *pfmdr1 *86Y and *pfcrt *76T alleles.

### *Pfdhfr *and *pfdhps*

PCR amplifications and sequencing of *pfdhfr *and *pfdhps *were successful in 218/240 (91%) and 231/240 (96%) samples, respectively. The prevalence of SNPs at codon 51, 59 and 108 of *pfdhfr *and codon 436, 437 and 540 of *pfdhps *are presented in Table 3. The *pfdhfr *alleles 108T, 16V and 164L were not observed.

**Table 3:** Prevalence of resistance associated single nucleotide polymorphisms in *pfdhfr *and *pfdhps *from southern Pakistan

	*Pfdhfr*				*Pfdhps*			
	
	n	51I	59R	108N	n	436A	437G	540E
**Karachi**	161	16 (0.1)	144 (0.89)	159 (0.99)	170	1 (0.006)	99 (0.58)	3 (0.02)
**Sindh**	48	2 (0.04)	44 (0.92)	47 (0.98)	51	0	13 (0.26)	0
**Baluchistan**	9	0	9 (1)	9 (1)	10	0	6 (0.60)	0
**Total**	218	18 (0.08)*	197 (0.90)^§^	215 (0.99)	231	1 (0.004)	118 (0.51)	3 (0.01)^†^

Allele proportions are shown in brackets. *Pfdhfr *alleles 108T, 16V and 164L were not observed.

* 3/218 had mix infection with *pfdhfr *51I and 51N alleles, ^§ ^5/218 had mix infection with *pfdhfr *59C and 59R alleles.

^† ^2/231 had mix infection with *pfdhps *540E and 540K allele

The *pfdhfr *double mutation 59R + 108N was found in 190/218 (87%) and 51I + 108N was found in 11/218 (5%) samples. The prevalence of *pfdhfr *double mutations 51I + 108N/59R + 108 was 201/218 (92%). The *pfdhfr *triple mutation (51I, 59R, 108N) occurred in 7/218 (3%) samples. The above mentioned *pfdhfr *triple mutation haplotype was found together with *pfdhps *437G in one sample. The combined *pfdhfr *(51I, 59R, 108N) and *pfdhps *(437G, 540E) quintuple mutation was found in one isolate from Karachi. The *pfdhfr *51, 59, 108,164 and *pfdhps *436, 437 and 540 haplotypes are presented in Table 4. Thirteen different *pfdhfr-pfdhps *haplotypes were identified.

**Table 4:** Prevalence of *pfdhfr- pfdhps *haplotypes in *P. falciparum *isolates from southern Pakistan.

*Pfdhfr *N51I, C59R, S108N, I164L *Pfdhps *S436A, A437G, K540E*		No. of isolates			
		
	No. of mutations	Karachi (n = 158)	Sindh (n = 47)	Baluchistan (n = 9)	Total (n = 214)
NCSI-SAK	0	-	1 (0.02)	-	1 (0.005)
N**RN**I-S**G**K	3	80 (0.51)	9 (0.19)	5 (0.55)	94 (0.44)
N**RN**I-SAK	2	55 (0.35)	33 (0.70)	4 (0.45)	92 (0.43)
**IRN**I-S**G**K	4	1 (0.006)	-	-	1 (0.005)
**IRN**I-S**GE**	5	1 (0.006)	-	-	1 (0.005)
**IRN**I-SAK	3	4 (0.03)	1 (0.02)	-	5 (0.02)
**I**C**N**I-SAK	2	5 (0.03)	-	-	5 (0.02)
**I**C**N**I-S**G**K	3	3 (0.02)	1 (0.02)	-	4 (0.02)
**I**C**N**I-S**GE**	4	2 (0.01)	-	-	2 (0.01)
NC**N**I-SAK	1	3 (0.02)	-	-	3 (0.01)
NC**N**I-S**G**K	2	2 (0.01)	2 (0.04)	-	4 (0.02)
NCSI**-A**AK	1	1 (0.006)	-	-	1(0.005)
NCSI-S**G**K	1	1 (0.006)	-	-	1(0.005)

*pfdhfr-pfdhps *haplotypes proportions are shown in brackets.

* Resistance associated alleles are indicated in bold.

## Discussion

This is the most comprehensive report characterizing resistance associated genetic polymorphisms in *P. falciparum *field samples collected in southern Pakistan. As such, the results bridge an important knowledge gap of the *P. falciparum *population in South Asia.

The 93% prevalence of *pfcrt *76T, essential for chloroquine resistance, is in line with results from neighbouring countries [16,31,32]. This data indicate high levels of *in vivo P. falciparum *chloroquine resistance in southern Pakistan. Moreover, the high *pfcrt *76T and moderately high (57%) *pfmdr1 *86Y prevalence also suggests high levels of tolerance/resistance to amodiaquine in the study area.

The high prevalence of the *pfdhfr *108N (99%) and 51I + 108N/59R + 108N (92%) in our study indicate that decreased susceptibility to sulphadoxine-pyrimethamine is widespread in Pakistan. However, only seven patients had infections with the triple *pfdhfr *resistance associated haplotype and only one patient was infected with *P. falciparum *that had the quintuple *pfdhfr *+ *pfdhps *haplotype associated with high grade sulphadoxine-pyrimethamine resistance. These results indicate that high grade resistance to sulphadoxine-pyrimethamine is not widespread and suggest that this drug is probably suitable for use with artesunate in southern Pakistan, as recommended by the National Malaria Control Programme. However, the occurrence of triple and quintuple mutant *P. falciparum *is of concern as widespread use of sulphadoxine-pyrimethamine as a partner to artesunate may rapidly select these haplotypes. Monitoring of *pfdhfr *and *pfdhps *resistance associated haplotypes is consequently of importance. Furthermore, artesunate + sulphadoxine-pyrimethamine *in vivo *efficacy urgently needs to be assessed. This is critical as efficacy studies conducted in Baluchistan (2001-2005) reported 56% treatment failure with sulphadoxine-pyrimethamine monotherapy [33]

Only one patient had a *P. falciparum *infection with two copies of *pfmdr1*, a finding that should be interpreted with caution. Increased *pfmdr1 *copy number has been associated with an increased risk for treatment failure after mefloquine monotherapy and artesunate-mefloquine therapy [26]. The low prevalence of *pfmdr1 *amplifications observed in this study suggests that both artesunate-mefloquine and artemether-lumefantrine would be efficacious in southern Pakistan. They may therefore represent potential future treatment alternatives to artesunate + sulphadoxine-pyrimethamine. Furthermore, the observed low prevalence of *pfcrt *76K and *pfmdr1 *86N provides supporting evidence of a probable high artemether-lumefantrine efficacy [20,24]

This data concur with recent results from a small study (n = 28) conducted in Khyber Pkhtunkhwan Province, Pakistan and previous data from India and Iran [34]. Furthermore, just as in northern India and Iran the *pfdhfr *A_16_N_51_**R**_59_**N**_108_I_164 _and A_16_**I**_**5**__1_**R**_59_**N**_108_I_164 _were the most common double and triple mutants found [35,36]. These results thus support a selective sweep of chloroquine and sulphadoxine-pyrimethamine resistant *P. falciparum *from southeast Asia via India to Pakistan and then on to Iran [37-40]

It should be underlined that a majority of patients (74%) presented with symptomatic malaria infection at the Aga Khan University Hospital that represents tertiary level of care. Some of these patients may have received anti-malarial treatment prior to enrolment in this study and the *P. falciparum *SNP proportions reported may, therefore, not be representative for the parasite population in southern Pakistan. However, the similarity of our results with those reported from neighbouring countries suggests that the results may be generalized.

## Conclusion

The results indicate high prevalence of *in vivo *resistance to chloroquine among *P. falciparum *in southern Pakistan, but high grade resistance to sulfadoxine-pyrimethamine does not appear to be widespread in the parasite population. This data thus support the recent change of first line therapy for uncomplicated *P. falciparum *malaria from chloroquine to artesunate + sulphadoxine-pyrimethamine. Continued anti-malarial drug resistance surveillance in Pakistan is essential.

## Competing interests

The authors declare that they have no competing interests.

## Authors' contributions

NKG performed DNA extractions and PCR genotyping, data entry, analysis and interpretation and drafted the first version of the report. JU designed and planned the study, performed data analysis and interpretation and wrote the report. MIV and SJ participated in PCR genotyping and data analyses. MAB and AM designed and planned the study, data analysis and interpretation and wrote the report. All authors read and approved the final manuscript.
